# Working agreement between the American Board of Radiology and The American Board of Medical Physics, Inc.

**DOI:** 10.1120/jacmp.v2i4.2599

**Published:** 2001-09-01

**Authors:** Lawrence E. Reinstein, Kenneth R. Hogstrom, Benjamin R. Archer

**Affiliations:** ^1^ The American Board of Medical Physics, Inc.

PACS number(s): 87.90.+y

To the Editor,

The American Board of Medical Physics (ABMP) wishes to share information regarding the recent adoption of a Working Agreement with the American Board of Radiology (ABR). As the Working Agreement deals with the certification of medical physicists, we believe it to be important to the clinical medical physicist. This information will be of interest to junior medical physicists, who are about to enter the process or are in the process of board certification, as well as medical physicists who hold ABMP certification.

On July 9, 2001, the ABMP unanimously approved and Chairman, Larry Reinstein, Ph.D. signed the Working Agreement on behalf of the ABMP. As the Working Agreement had been previously approved by the ABR, it became effective immediately. The ABMP presented an announcement at the meeting of the Board of Directors of the American Association of Physicists in Medicine (AAPM) on July 26, 2001 in Salt Lake City, UT. The ABR also presented an announcement, and the two announcements and Working Agreement were officially received into minutes. We believe that these documents should be made readily available to all medical physicists by publication in the archival literature.

The text of the ABMP announcement is as follows:

**ABMP Announcement of ABR‐ABMP Working Agreement at AAPM Board of Directors Meeting**

July 26, 2001

The American Board of Medical Physics (ABMP) is pleased to announce that it has entered into a Working Agreement with the American Board of Radiology (ABR) effective July 9, 2001. This Agreement was approved unanimously by both the ABMP and ABR, and it creates an environment in which the two medical physics certification boards can work closely together for the benefit of medical physics. The ABMP believes this Agreement will unify the medical physics community and eliminate the confusion among entry‐level medical physicists resulting from the two‐board system. Furthermore, it will strengthen the certification process in traditional fields of medical physics by uniting the resources of both boards.

Under the terms of the agreement, the ABMP will continue to exist but will no longer offer to new candidates, certification examinations in the traditional fields of medical physics (namely radiation oncology physics and diagnostic imaging physics). The ABMP will focus its future certification efforts in the non‐traditional fields of medical physics, currently magnetic resonance imaging (MRI) physics and medical health physics, and in the development of subspecialty certifications, such as cardiovascular physics and neuro‐irradiation physics. The ABMP will also retain its ability to rapidly expand into areas of new certification to meet changing needs in the medical physics environment.

Although the ABMP will no longer participate in certification in the traditional areas for new candidates, the two boards will participate jointly in the certification of medical physicists. The composition of the certification oversight committees will be equally divided among members appointed by each board. The ABMP will recommend three voting members to be appointed to the ABR Radiological Physics Examination Committee, and the ABR will recommend an equal number of voting members for appointment to an ABMP MRI Physics Committee.

The Agreement allows candidates currently engaged in the ABMP certification examination process the option of completing their examination with the ABMP or the ABR. ABMP certified diplomates may apply for, and receive, an ABR letter of certification equivalence. This letter allows them the opportunity to become members of the American College of Radiology (ACR) and to be recognized as equivalent to the ABR diplomates in all guidelines, standards, regulations, and privileges of scientific, professional, and regulatory bodies. The ABR will issue full certification and listing in the American Board of Medical Specialties to any ABMP diplomate having an ABR letter of certification equivalence and having satisfied all conditions of its future Maintenance of Certification program.

The ABMP invites and expects the American Association of Physicists in Medicine (AAPM) to become a sponsoring organization. In the near future, the ABMP will modify its bylaws and develop future goals to meet its new strategic direction and role as established with this Agreement. At the AAPM Board of Directors meeting during the 2001 annual meeting of the Radiological Society of North America (RSNA), the ABMP will invite the AAPM to become a sponsoring organization and will request moderate funding assistance for its new mission.

The ABMP wishes to recognize the ABR physics trustees for their persistence and constructive demeanor throughout the negotiating process, which began at last year's AAPM annual meeting. We also thank the AAPM and its president, Charles Coffey, for hosting the meetings of the two Boards' representatives and for continued encouragement and support throughout the process.

This Agreement is an extraordinary event in the history of medical physics. It is the sincere hope of the ABMP that the spirit of cooperation and goodwill that prevailed during these negotiations will infuse the medical physics community, so that we can all work together toward the advancement of our profession.

The text of the ABR‐ABMP Working Agreement is as follows:

WORKING AGREEMENT

AMERICAN BOARD OF RADIOLOGY

and

THE AMERICAN BOARD OF MEDICAL PHYSICS, INC.

June 4, 2001

This agreement is effective as of this $9^{\text{th}}$ day of July, 2001 by and between the “parties” herein defined as the American Board of Radiology (ABR), a not‐for‐profit corporation with principal offices located at 5255 E. Williams Circle, Suite 3200, Tucson, Arizona 85711‐7409 U.S.A. and The American Board of Medical Physics, Inc. (ABMP), a not‐for‐profit corporation with its principal office c/o Dr. Herbert Mower, Lahey Hitchcock Medical Center, 41 Mall Road, Burlington, MA 01805.

WHEREAS ABR and ABMP each certify medical physicists;

WHEREAS ABR and ABMP mutually agree that the process of certification of medical physicists should be defined and administered by board‐certified medical physicists;

WHEREAS ABR and ABMP mutually agree that having a single certification board for each field and subspecialty of medical physics will reduce confusion of medical physicists and the public;

WHEREAS ABR and ABMP agree that having a single certification board for each field and subspecialty will increase efficiency of the process of certification of medical physicists;

WHEREAS ABR and ABMP also agree that having a single certification board for each field and subspecialty will increase the stature of certification to individuals obtaining it; and

WHEREAS ABR and ABMP mutually agree to work together from the date of this agreement forward to achieve and maintain a single certification board for each field and subspecialty of medical physics;

NOW, THEREFORE, for and in consideration of the mutual covenants set forth in this agreement, it is agreed by and between the parties as follows:

ARTICLE 1 ‐ Purpose of the agreement

1.1 This agreement embodies and describes an intent of ABR and ABMP to work cooperatively to achieve shared objectives with regard to certification in medical physics.
1.2 Specific terms of cooperation described in this agreement represent the initial objectives of cooperation between the parties; cooperation will continue towards the goal of continually improving the certification of medical physicists after the initial objectives have been achieved.
1.3 The parties acknowledge that the spirit of cooperation evidenced in this agreement represents a long‐sought goal that supports the public interest and the professional standards of medical physics.

ARTICLE 2 ‐ Certification of new candidates in *traditional fields* of medical physics

2.1 *Traditional fields* of medical physics include the areas of radiation therapy physics (therapeutic radiological physics), diagnostic radiology physics (diagnostic radiological physics), and nuclear medicine physics (medical nuclear physics).
2.2 After the July 21, 2001 examination, the ABMP will no longer offer the Part I Written Examination for new candidates in the *traditional fields* of medical physics. All *new* candidates for certification in these areas must apply to the ABR.

ARTICLE 3 ‐ Certification of candidates in *traditional fields* of medical physics who are currently in the process of obtaining ABMP certification

3.1 Candidates engaged in the ABMP certification process after the July 22, 2001 ABMP examinations may continue the ABMP certification process.
3.2 Candidates engaged in the ABMP certification process, who have passed Parts I or II, may transfer into the corresponding stage of the ABR certification process.
3.3 ABMP oral examination candidates who have conditioned in the one or more areas and transfer to the ABR oral examination process must take the full ABR oral examination.

ARTICLE 4 ‐ Certification in *non‐traditional* fields of medical physics

4.1 *Non‐traditional fields* of medical physics include independent areas of medical physics certification other than *traditional fields* of medical physics.
4.2 The ABR will not examine medical physicists in *non‐traditional fields* of medical physics in which the ABMP offers certification by examination, and similarly the ABMP will not examine medical physicists in *non‐traditional* fields of medical physics in which the ABR offers certification by examination.
4.3 The ABMP presently certifies medical physicists in three *non‐traditional fields* of medical physics: hyperthermia physics, medical health physics, and MRI physics. In exception to 4.2 the ABR may offer certification by examination in MRI physics five years after the effective date of this agreement.
4.4 The ABMP will provide the ABR with equal participation in its *non‐traditional* certification examination in MRI physics. Equal participation is defined as providing the ABR with equal representation of voting members on an ABMP committee, which is responsible to the ABMP for conducting examination for certification. The ABR members will be selected by the ABMP from nominees submitted by the ABR. The ABR will submit 2 nominees for each member to be selected by the ABMP.

ARTICLE 5 ‐ Certification in medical physics *subspecialities*

5.1 *Subspecialty* certification examinations shall require certification by the ABR or by the ABMP in a *traditional or non‐traditional field* of medical physics.
5.2 The ABMP retains the right to develop *subspecialty* certification examinations (e.g. in cardiovascular or neuro‐irradiation physics). The ABR will not certify in *subspecialties* of medical physics.

ARTICLE 6 ‐ Certification equivalency for ABMP diplomates

6.1 Medical physicists certified by the ABMP in a *traditional field* of medical physics will, upon written request, receive a letter of certification equivalence from the ABR stating that ABMP certification is equivalent to ABR certification in the same field. The letter of certification equivalence will be available for 5 years from the date of this agreement.
6.2 The ABR acknowledges that ABMP diplomates with a letter of certification equivalence in a *traditional field* of medical physics should be recognized as equivalent to ABR diplomates (in the same *traditional field*) in all guidelines, standards, regulations, and privileges of scientific, professional, and regulatory bodies.
6.3 The ABR has been informed by the American College of Radiology (ACR) that medical physicists receiving a letter of certification equivalence will be eligible for membership in the ACR on the same basis as medical physicists certified by the ABR (see Attachment A).

ARTICLE 7 ‐ Recertification of ABMP diplomates

7.1 Each medical physicist certified by the ABMP will be eligible to participate in the recertification program of the ABMP.
7.2 Those ABMP diplomates who attain the ABR letter of certification equivalence after December 31, 2002 will be required to participate in the ABR's Maintenance of Certification program in order to maintain the letter of certification equivalence.
7.3 Any ABMP diplomate with a letter of certification equivalence is eligible for the ABR's Maintenance of Certification program. If all conditions for maintenance of certification are satisfied, these physicists will be awarded an ABR certificate and placed in the listings of the American Board of Medical Specialties.

ARTICLE 8 ‐ ABR Radiological Physics Examination Committee

8.1 Membership: Consistent with Article VIII, Section 5 of the ABR Bylaws, this agreement establishes that the voting members of the Radiological Physics Examination Committee shall consist of the three ABR Physics Trustees and an equal number of members from the ABMP. The ABMP members will be selected by the ABR from nominees submitted by the ABMP. The ABMP will submit 2 nominees for each member to be selected by the ABR.

ARTICLE 9 ‐ Funding

9.1 Parties of this agreement recognize that termination by the ABMP of certification of medical physicists in *traditional fields* of medical physics will cause financial hardship to the ABMP for a 3‐year transition period (July 1, 2002 to June 30, 2005).
9.2 The ABR agrees to provide transition compensation to the ABMP over three years for lost revenue caused by this termination of certification.
9.3 Compensation shall be as follows: a first payment of $15,000 by September 30, 2002; a second payment of $15,000 by September 30, 2003; and a third and final payment of $15,000 by September 30, 2004.
9.4 No other payment or compensation for any purpose shall be paid by the ABR to the ABMP.

ARTICLE 10 ‐ Continued Cooperation

10.1 The intent of both parties is to continue to cooperate to improve the *process* of certification of medical physicists after the specific terms of the agreement have been satisfied.
10.2 The intent of both parties is to continue to cooperate to improve the *stature* of certification of medical physicists. This shall include, but is not limited to, the ABR and ABMP encouraging the ACR, American College of Medical Physics (ACMP), American Association of Physicists in Medicine (AAPM) and other similar professional organizations' inclusion of ABR and ABMP medical physics certification in appropriate standards and definitions of medical physicists.
10.3 The intent of both parties is to continue to cooperate to improve the *unity* of the process of certification of medical physicists.

(The Working Agreement was signed by Robert R. Hattery, M.D., President of the American Board of Radiology on June 10, 2001 and by Lawrence E. Reinstein, PhD., Chairman of The American Board of Medical Physics, Inc. on July 9, 2001.)

Medical physicists who are ABMP diplomates or are in the process of ABMP certification examination will receive a mailing that includes the material presented here as well as a Frequently Asked Questions document that will continue to evolve and will be available on the American College of Medical Physics web site (www.acmp.org).

Thank you for allowing the ABMP to share this information with practicing medical physicists through the JACMP.

